# Transfer Function of Macro-Micro Manipulation on a PDMS Microfluidic Chip

**DOI:** 10.3390/mi8030080

**Published:** 2017-03-04

**Authors:** Koji Mizoue, Kaoru Teramura, Chia-Hung Dylan Tsai, Makoto Kaneko

**Affiliations:** Department of Mechanical Engineering, Osaka University, Suita 565-0871, Japan; mizoue@hh.mech.eng.osaka-u.ac.jp (K.M.); teramura@hh.mech.eng.osaka-u.ac.jp (K.T.); mk@mech.eng.osaka-u.ac.jp (M.K.)

**Keywords:** cell manipulation, transfer function, microfluidic channel, macro actuator

## Abstract

To achieve fast and accurate cell manipulation in a microfluidic channel, it is essential to know the true nature of its input-output relationship. This paper aims to reveal the transfer function of such a micro manipulation controlled by a macro actuator. Both a theoretical model and experimental results for the manipulation are presented. A second-order transfer function is derived based on the proposed model, where the polydimethylsiloxane (PDMS) deformation plays an important role in the manipulation. Experiments are conducted with input frequencies up to 300 Hz. An interesting observation from the experimental results is that the frequency responses of the transfer function behave just like a first-order integration operator in the system. The role of PDMS deformation for the transfer function is discussed based on the experimentally-determined parameters and the proposed model.

## 1. Introduction

There are various situations where cell manipulation is required in microfluidic applications [1]. The manipulation speed and resolution have been previously achieved up to 130 Hz and 240 nm as moving a micro object in a simple harmonic motion (SHM) on a microfluidic chip [2]. In order to further improve the manipulation speed and accuracy, as well as for a better understanding of the system, it is important to know the transfer function of the system. With such a transfer function, a controller can be customized and optimized based on the system characteristics. 

**Figure 1:**
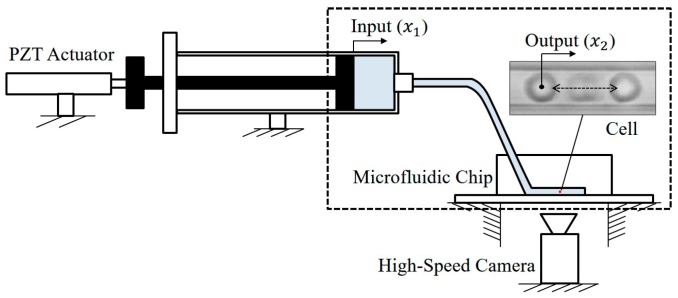
An illustrative diagram demonstrates how cell manipulation is controlled by a macro actuator outside the microfluidic chip.

Figure 1 illustrates a diagram of manipulating a micro object, for example, a red blood cell, in a microfluidic chip using a macro actuator. The object is suspended by the fluid in the microchannel, and it moves with the fluid flow. The macro actuator controls the syringe pump for producing different rates and directions of the flow inside the channel. There are advantages of using a macro actuator instead of an on-chip micro actuator for such a manipulation. One of the advantages is that macro actuator, such as the piezoelectric (PZT) actuator shown in Figure 1, or a linear slider, are commercially available so that it is very convenient to implement them into a manipulation system [3,4]. Another advantage is that the macro actuator can be repeatedly used since it is separated from the microfluidic chip, while a micro actuator is usually fabricated on the chip, and it is difficult to be re-used [2,5].

However, the manipulation using a macro actuator is challenging because even a slight motion from the actuator may result in a very large displacement for the target object. This is due to the large ratio between the micro and macro cross-sectional areas, and a reduction mechanism is necessary for making this possible. Fortunately, polydimethylsiloxane (PDMS), one of the most common materials for making a microfluidic system, is embedded with a natural reduction mechanism from its deformable characteristic [6].

The transfer function and its frequency responses are experimentally investigated in this work. An open-loop control system is employed for determining the transfer function of the system. Different frequencies of sinusoidal inputs are applied to the PZT actuator as the inputs of the transfer function, while the motions of micro-objects are tracked as the outputs. The maximum frequency of the input signal is up to 300 Hz SHM, which is more than double that of previous works [2,7]. The gains and phase at different frequencies are determined using a fast Fourier transform (FFT). It is found that the system response is very similar to an integration operator that the gain is linearly decreased in Bode plots while the phase is constantly around −90°. The experimental results are applied back to the derived model and a very well fit is obtained. Finally, the system parameters are identified and discussed.

In summary, we focus on directly identifying the transfer function from the experimental inputs and outputs. A mechanical model of the system is proposed, and the experimental results are discussed with the model. The rest of this paper is organized as follows. After briefly reviewing the related works in Section 2, a mechanical model for the macro-micro manipulation system is proposed in Section 3. The experimental method and results are presented in Section 4 and Section 5. The results are discussed in Section 6. Finally, concluding remarks are summarized in Section 7.

## 2. Related Works

Various approaches have been developed for cell manipulation, for example, using flow control in a microfluidic channel [2,6,8,9], optical tweezers [10,11], micro grippers [12,13,14,15], electrical or magnetic force [16], and acoustic trapping [17,18,19]. The combination of a high-speed pump and a high-speed vision is often used together for high-speed manipulation in a microfluidic channel. For example, Chen et al. performed high-speed cell sorting of single bacterial cells by a PZT pumping [20]. The manipulation is especially important for active cell assessments. For example, Monzawa et al. introduced an actuation transmitter for cell manipulation and evaluation [2]. Sakuma et al. applied a fatigue test to human red blood cells by imparting periodical mechanical stress [8]. Murakami et al. investigated the shape recovery of a cell by controlling different loading time in a constriction [21]. The throughput and stability of such active assessments directly depend on the manipulation speed and resolution. While the frequency characteristics under such closed-loop manipulation systems were previously discussed [2,6], the open-loop transfer function is important to know for designing a faster and more accurate cell manipulation system. To the best of our knowledge, there have been no works discussing the transfer function of the open-loop PDMS microfluidic channel, and this is the first work that exploits the true nature of the macro-micro manipulation on a PDMS chip.

## 3. Modeling of Transfer Function with a PDMS Microfluidic Channel

**Figure 2:**
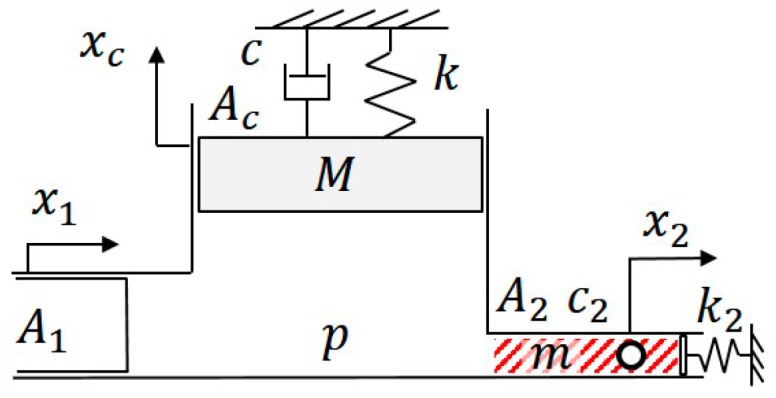
The proposed mechanical model for describing the relation between the movement between a macro actuator ($x_{1}$) and a micro object ($x_{2}$). The model describes the dashed box area shown in Figure 1.

Figure 2 shows the model of the cell manipulation system where $x_{1}$ and $x_{2}$ are the input, the PZT actuator motion, and the output, the micro object motion of the system. $M,\;k,\;c,\;A_{c}$, and $x_{c}$ are the equivalent mass, stiffness, damping, cross-sectional area, and displacement of a virtual element which represents an equivalent piston motion for the deformation of PDMS, tube, and other deformable parts from the actuator to the chip. $A_{1}$ and $A_{2}$ are the cross-sectional areas of the PZT-actuated pump and the microchannel. $P,\;k_{2},\;c_{2}$, and m are the fluid pressure at the macro-micro junction, the stiffness of PDMS at the channel outlet, the fluid viscosity, the mass of fluid in the channel, respectively. Since this model describes a macro-micro manipulation system, the value of $A_{1}/A_{2}$ is in the order of $10^{6}$. The equation of fluid continuity and the equations of motion can be written as:

(1) $$A_{1}x_{1}=A_{c}x_{c}+A_{2}x_{2}$$

(2) $$pA_{c}=M\;{\ddot{x}}_{c}+c{\dot{x}}_{c}+kx_{c}$$

(3) $$pA_{2}=m\;{\ddot{x}}_{2}+c_{2}{\dot{x}}_{2}+k_{2}x_{2}$$

where ${\dot{x}}_{c}$, ${\dot{x}}_{2}$ and ${\ddot{x}}_{c}$, ${\ddot{x}}_{2}$ are the first and second time derivatives of $x_{c}$ and $x_{2}$, respectively. The transfer function can be derived in the frequency domain after Laplace transform as shown in Appendix A and the resulting function is:

(4) $$\frac{X_{2}}{X_{1}}=\;\alpha \frac{s^{2}+2\zeta_{1}\omega_{1}s+\omega_{1}^{2}}{\;s^{2}+2\zeta_{2}\omega_{2}s+\omega_{2}^{2}}$$

where $X_{1}$, $X_{2}$, and s are the Laplace transforms of $x_{1}$ and $x_{2}$, and a complex number representing the input frequency. The coefficients $\alpha ,{\;\zeta }_{1},\;\zeta_{2},\;\omega_{1},\;\omega_{2}$ are defined by physical parameters of the system and are:

$$\alpha =\frac{A_{1}M}{A_{2}\left[M+{\left(\frac{A_{c}}{A_{2}}\right)}^{2}m\right]}$$

$$\zeta_{1}=\frac{c}{2\sqrt{Mk}}$$

$$\omega_{1}=\sqrt{\frac{k}{M}}$$

$$\zeta_{2}=\frac{c+{\left(\frac{A_{c}}{A_{2}}\right)}^{2}c_{2}}{2\sqrt{\left[M+{\left(\frac{A_{c}}{A_{2}}\right)}^{2}m\right]\left[k+{\left(\frac{A_{c}}{A_{2}}\right)}^{2}k_{2}\right]}}$$

$$\omega_{2}=\sqrt{\frac{k+{\left(\frac{A_{c}}{A_{2}}\right)}^{2}k_{2}}{M+{\left(\frac{A_{c}}{A_{2}}\right)}^{2}m}}$$

the transfer function in Equation (4) is for displacement-based pumps while the pressure-based pumps would be much simplified and Equation (3) will be the only governing equation. However, a pressure-based pump only measures the pressure at the pump outlet. The actual pressure in a microfluidic system remains unknown. As a result, the pressure value of such a pressure-based pump is not applicable for the proposed model.

## 4. Experiments

**Figure 3:**
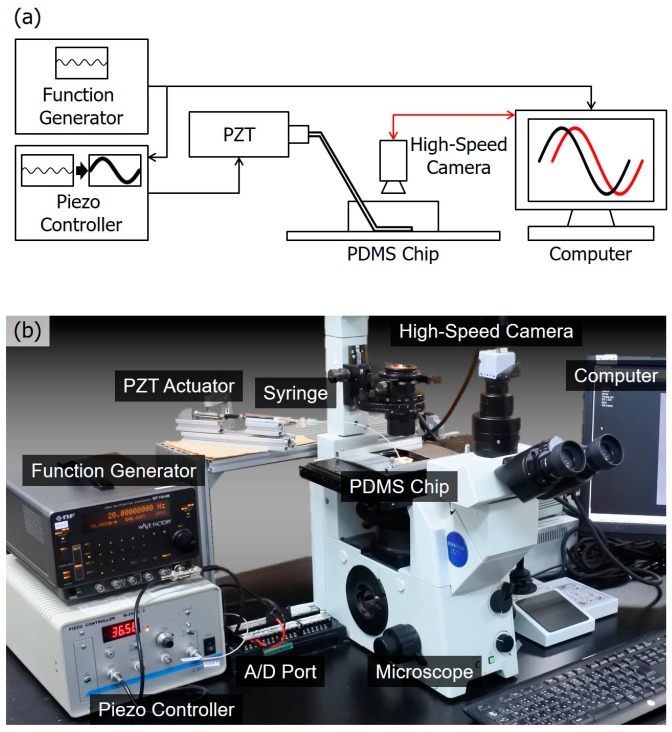
An overview of the experimental system. (**a**) A diagram of the system setup. The motion of the piezoelectric (PZT) actuator is controlled by a sinusoidal wave generated from a function generator while the motion of microbeads is recorded by a high-speed camera. The two motions are synchronized on the computer for analysis; (**b**) a photo of the actual system setup.

Figure 3a shows a diagram of the overall system setup. The macro PZT actuator is directly controlled by a sinusoidal function from a function generator, and the offset and a peak-to-peak amplitude of the signal are 1 V and 0.4 V, respectively. The 0.4 V voltage would generate a peak-to-peak displacement of 1.6 µm at the tip of the PZT actuator. The input voltage is also sent to the computer through an analog-digital (A/D) port for synchronization of time in later analysis. The computer controls a high-speed camera for recording the motion of the microbeads that are suspended in the fluid in the channel. The sinusoidal motion of the PZT actuator and the microbeads’ motion are taken as the input and output of the transfer function. Both the input and output are recorded on the same computer with timestamps for determining the phase lag.

Figure 3b shows a photo of the actual system. The system is composed of a PDMS chip including a microfluidic channel, a digital function generator (WF1944B, NF Corp., Yokohama, Japan), a PZT actuator (PSt150/5/40, Syouei System, Co., Ltd., Tokyo, Japan), a PC Card (CSI-360116, Interface Co., Hiroshima, Japan), a microscope (IX71, Olympus Co., Tokyo, Japan), a high-speed camera (IDP, Photron Co., Tokyo, Japan), and a computer. The PDMS chip is molded by a mold patterned by photoresist (SU8-3005, Nippon Kayaku Co., Ltd., Tokyo, Japan) fabricated with a standard photolithography process [2]. The mechanical characteristics depend on the degree of crosslinking, which is determined by the ratio between PDMS and the curing agent while making the chip [22]. In this particular case, the ratio is 1:9 for the curing agent and PDMS, respectively. The height and width of the microfluidic channel are 3 µm and 100 µm, respectively. The recording rate of the camera is 1000 frames per second (fps). Polymeric microbeads of diameters of 1 µm are used as manipulation targets (4009A, Thermo Fisher Scientific Inc., Waltham, MA, USA). An advantage of using microbeads instead of biological cells is that we can neglect the possible effect coming from object properties, such as shape changing.

## 5. Results

### 5.1. Input-Output Relation

**Figure 4:**
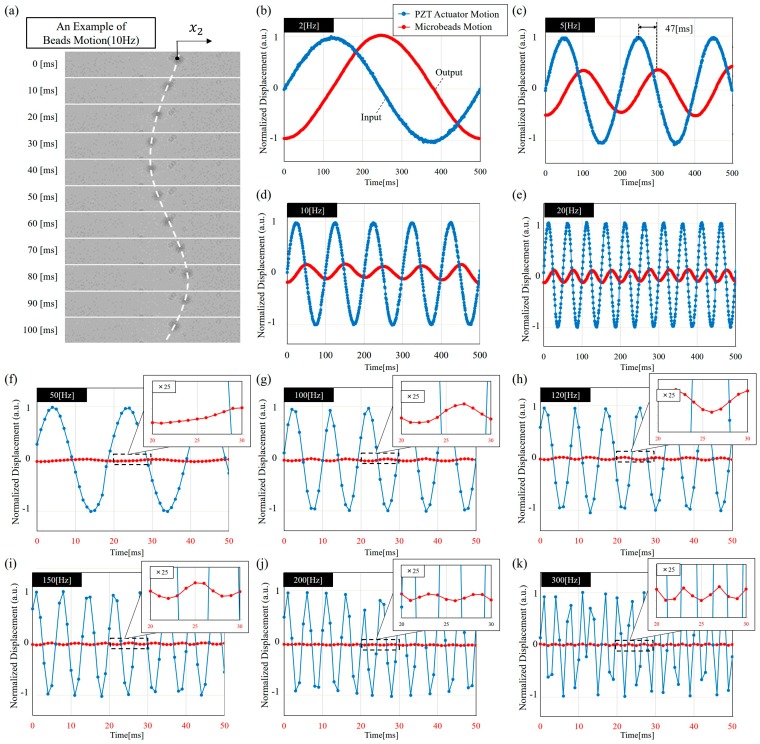
The experimental results. (**a**) A combination of multiple snapshots showing a microbead moving in a sinusoidal function at 10 Hz; (**b**)–(**k**) Examples of input-output relations at the frequencies of 2 Hz, 5 Hz, 10 Hz, 20 Hz, 50 Hz, 100 Hz, 120 Hz, 150 Hz, 200 Hz, and 300 Hz, respectively. For the convenience of reading, the time range is 0 ms to 500 ms in (**b**)–(**e**) and 0 ms to 50 ms in (**f**)–(**k**).

Figure 4 shows examples of the results obtained from the experiments. Figure 4a shows an example of microbeads motion under 10 Hz actuation from PZT actuator. The position of the microbead is calculated as the centroid of the microbead as $x_{2}$ indicated in Figure 4a. Figure 4b–k are examples of the normalized motion under 2 Hz, 5 Hz, 10 Hz, 20 Hz, 50 Hz, 100 Hz, 120 Hz, 150 Hz, 200 Hz, and 300 Hz, respectively. Figure 4b–k includes both the input and output signals which are the motions of PZT actuator and a microbead at each frequency. Since the magnitudes of the inputs and outputs in this setup is about 30 times different, the input and output plots are normalized separately with respect to the signal at 2 Hz in Figure 4b. Arbitrary units (a.u.) are used for letting the data range be from −1 to 1. Figure 4f–k additionally include a zoomed-in view for showing small amplitudes of microbead motion. In Figure 4b–k, clear phase lags between the input and output can be observed. For example, the lag of 47 ms can be visually measured by the signal peaks in Figure 4c. FFT is employed for analyzing the input-output relation for more systematic measurements.

**Figure 5:**
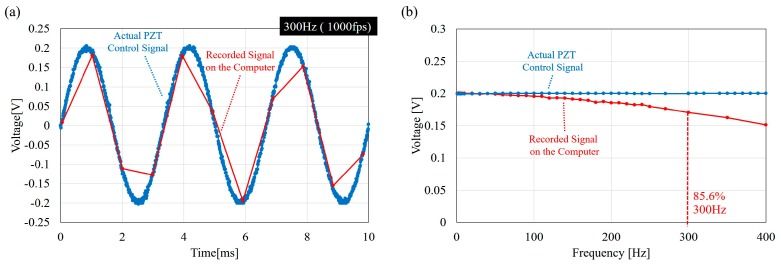
The signal distortion due to the limit of temporal resolution in C program. (**a**) An example of distorted signal at 300 Hz where the input signal is generated at the rate of 100 kHz while the sampling rate on the computer is only 1 kHz; and (**b**) the comparison of the sinusoidal amplitudes on the PZT actuator and on the computer. The computer side remains only 85.6% at 300 Hz.

Figure 5 shows the actual PZT control signal and the signal recorded by the computer through the A/D port. Distortion of the record signals can be found in the recorded data due to the computer recording rate. Input signal distortion and time delay plays a crucial role in system identification and, thus, are specially addressed here. The sampling time for the function generator and the computer in this work are 100 kHz and 1 kHz, respectively. The signal distortion is due to the limit of the timer in the standard C timer function “*timeSetEvent*” where the resolution is limited to a millisecond. As a result, although the PZT actuator is controlled by a nearly perfect sinusoidal wave from the function generator, the signals received at the computer for synchronization and analysis are distorted at high frequencies, such as 300 Hz, as shown in Figure 5a. Figure 5b shows the comparison between the average amplitude of the input signals on the PZT actuator and on the computer. The distortion in terms of amplitude is not significant for the frequencies below 100 Hz because the amplitudes are closely overlapped in this region in Figure 5b. The amplitude gradually reduces with a further increase of frequency beyond 100 Hz. When the input frequency reaches 300 Hz, only 85% of the amplitude remains. The recordings on the computer are necessary for the signal synchronization in the experiments because the input and output signals are from two independent devices, which are the function generator and the high-speed camera. Therefore, the distortion is considered as a necessary sacrifice for the synchronization, and a better synchronization approach is needed for even higher frequencies.

**Figure 6:**
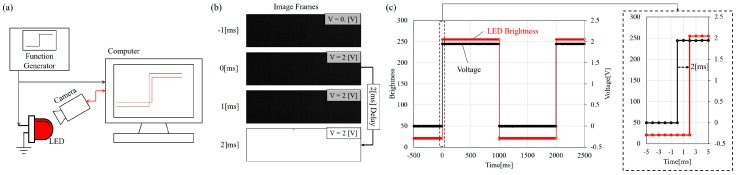
The measurement of time delay on the computer. (**a**) The diagram of the setting; (**b**) the signal sent to the light-emitting diode (LED) at 0 ms while the high-speed camera captured the LED response at 2 ms; and (**c**) the plot of the input and output in time delay test. The delay is 2 ms.

Figure 6 shows system delay test for measuring the time delay between A/D signal receiving and high-speed camera recording. The setting of the test can be found in Figure 6a where a function generation is directly connected to a fast-response light-emitting diode (LED). Figure 6b shows the timing of signal change and recorded image frames while Figure 6c is the results plotted in the graph. An average delay of 2 ms is consistently observed. This means that the actual phase lag would be overestimated by 2 ms if such a delay is not considered.

The 2 ms may be negligible at lower frequencies but is significant when the frequency goes over 100 Hz. For example, when the input frequency is 300 Hz, the 2 ms becomes −216° phase shift considering the period of 300 Hz signal is only 3.3 ms.

### 5.2. The Gain and Phase Characteristics

**Figure 7:**
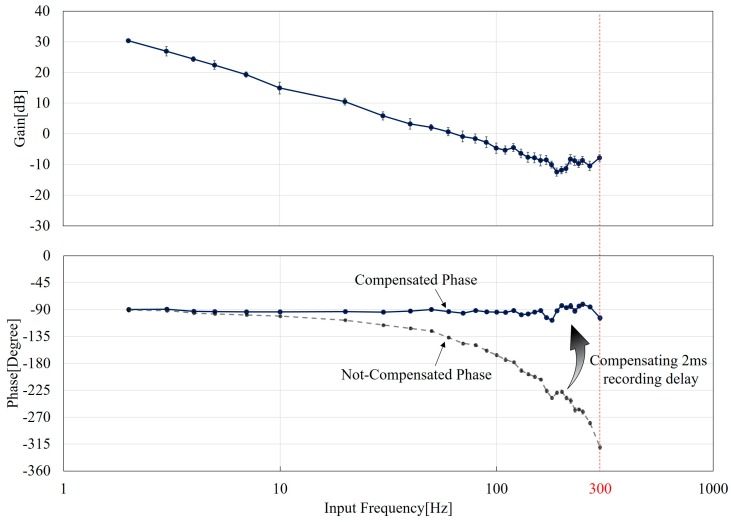
The Bode plots from experimental results. The upper and lower plots are the gain and phase response, respectively. The delay of 2 ms in the recording system is essential for the phase. Both before and after the compensation plots are shown.

Figure 7 shows the Bode diagram of the manipulation system where the upper and lower plots are the gain and phase characteristics with respect to different frequencies. The values of the gain and phase are determined using FFT, and the details of how the values are obtained are explained in Appendix B. The gain decreases with the increase of the input frequency while fluctuations can be seen at high frequencies over 100 Hz. The fluctuation may be due to the distortion of the input as shown in Figure 5. Both the phase responses with and without the compensation are plotted in the phase graph as the blue and gray curves in Figure 7. The 2 ms time delay is as expected significantly affecting the phase response while the compensated phase results are remaining constant around −90°. Surprisingly, the frequency responses of the transfer function in Figure 7 looks similar to an integration operator, α/s. That is, the gain constantly decreases along a −20 dB line and the phase is maintained at −90°. It is very interesting to see a simple first-order response of the system even though the general response in Equation (4) is second-order. Further discussions and comparisons on this result are in the following section.

## 6. Discussions

The system can be simplified by considering the similarity to an integrator as stated at the end of Section 5 and the shape of Bode plots shown in Figure 7. By choosing the gain value of 30 at 2 Hz along with the assumption of −20 dB decrease according to Figure 7, the intersect on the *y*-axis at very low frequency (s→0) can be estimated as 36. Thus, we can obtain a simplified transfer function of the PDMS-based macro-micro manipulation system as:

(5) $$G\left(s\right)\sim \frac{63}{s}$$

where s is a complex number representing the input frequency as in Equation (4). Equation (5) indicates that the transfer function is like the input and output of an integration operator in the control system. The gain reduces when the frequency goes high, while the phase shift is consistently at −90°.

While Equation (5) shows a visually-identified transfer function based on the experimental results in Figure 7, we also applied our model for a more generalized discussion here. A curve fitting is employed to identify the system parameter in Equation (4) from the experimentally-obtained gain data. The curve fitting on the gain is optimized by the least-square algorithm for the best fit. The fitting result gives:

$$\alpha =0.01872,{\;\omega }_{1}=1321,{\;\omega }_{2}=0.36,\;\zeta_{1}=7.7,\;{\;\zeta }_{2}=104.8$$

The coefficient of determination ($R^{2}$) of the fit is 0.9993, which reflects a very good fit. Finally, we have the transfer function with identified parameters as:

(6) $$G\left(s\right)=0.01872\frac{s^{2}+20261.5s+1745041}{\;s^{2}+76.3s+0.13}$$

**Figure 8:**
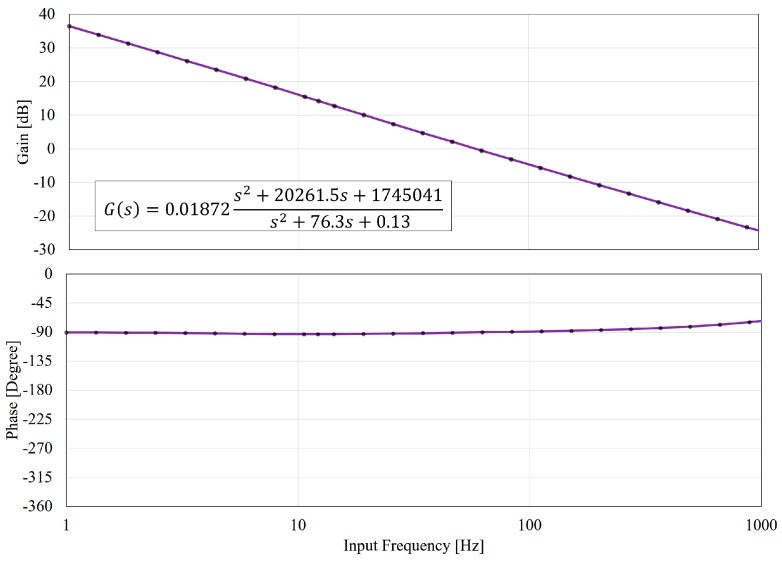
The Bode plots based on the proposed model in Equation (6) and the curve fitting on the gain response. Both the gain and the phase match well to the experimental analysis in Figure 7. The spectrum is further extended to 1000 Hz for the prediction of the system response at higher frequencies.

Figure 8 shows the Bode diagram of Equation (6) with an extended frequency range up to 1000 Hz, which is very challenging to achieve for physical systems. The phase in Figure 8 also well fits with the experimental results in Figure 7 although the least-square fit only applies to the gain results. According to Figure 8, the gain of the PDMS-based manipulation system consistently decays. The phase lag is roughly kept at −90° but is gradually increasing around 1000 Hz. It can be interpreted as that when the input frequency is high, the deformable part, such as springs and dampers in Figure 2, cannot timely respond to the high frequency. In other words, the channel wall can be seen as getting stiffer at high frequencies, and as a result, the phase lag reduces. It is physically reasonable since such a mass-spring-damper system often behaves like a low-pass filter.

An interesting insight of the PDMS system can be found by comparing Equations (4) and (5). The general transfer function in Equation (4) is a second-order system with two zeros and poles while the Equation (5) is a very simple first-order system with a pole at zero frequency. That means the dominant terms that govern the transfer function are only the $\left(\frac{A_{1}}{A_{2}}\right)k$ in the numerator and $\left[c+{\left(\frac{A_{c}}{A_{2}}\right)}^{2}c_{2}\right]s$ in the denominator. It demonstrates the inertia for such a macro-micro manipulation system is not significant. The physical meaning of the integration operator can be realized as a different view of the Hagen-Poiseuille equation [23], which can be simplified as:

(7) $$x_{2}=\int \frac{\Delta P_{\mathrm{ch}}}{\kappa }dt$$

where $\Delta P_{\mathrm{ch}}$ and κ are the pressure drop across the microfluidic channel and the coefficient including fluid viscosity and channel dimensions.

## 7. Conclusions

This paper reveals the frequency characteristics between a macro actuator and a micro object in a PDMS microfluidic channel and aims at improving cell manipulation systems at high speed. Both the theoretical model and experimental validations are presented. The gain and phase of the transfer function are obtained. The theoretical model can fit well with the experimental results, and the system parameters are identified. According to the experimental results, the PDMS microfluidic device works like an integrator, 1/s, and it can be understood as another form of the Hagen-Poiseuille equation. This work provides the physical insight of the PDMS microfluidic chip and can contribute to on-chip manipulation systems.

## Figures and Tables

**Figure A1:**
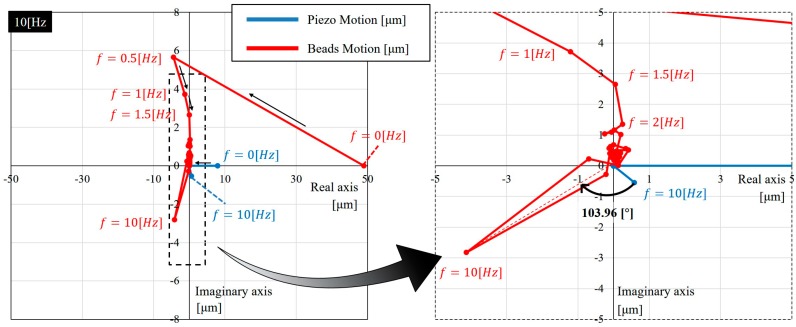
An example of how the gain and phase are determined using fast Fourier transform (FFT) and its results.

## Appendix A

The derivations and general discussion on the theoretical modeling are presented in this appendix. Equations (1)–(3) can be converted from time domain to frequency domain by Laplace transform as:

(A1) $$A_{1}X_{1}=A_{c}X_{c}+A_{2}X_{2}$$

(A2) $$pA_{c}=M\;s^{2}X_{c}+csX_{c}+kX_{c}$$

(A3) $$pA_{2}=m\;s^{2}X_{2}+c_{2}sX_{2}+k_{2}X_{2}$$

where $X_{1}$, $X_{2}$, and s are the Laplace transforms of $x_{1}$ and $x_{2}$, and a complex number representing the input frequency. By solving the algebra equations in Equations (A1) to (A3), the transfer function between input $X_{1}$ and output $X_{2}$ is then obtained as follows:

(A4) $$\frac{X_{2}}{X_{1}}=\frac{A_{1}}{A_{2}}\frac{Ms^{2}+cs+k}{\left[M+{\left(\frac{A_{c}}{A_{2}}\right)}^{2}m\right]s^{2}+\left[c+{\left(\frac{A_{c}}{A_{2}}\right)}^{2}c_{2}\right]s+\left[k+{\left(\frac{A_{c}}{A_{2}}\right)}^{2}k_{2}\right]}$$

Equation (A4) shows the transfer function of the macro-micro manipulation system based on the model in Figure 2. To further understand the physical meaning of the function, we firstly consider the two extreme cases of the input frequency s as s→0 and s→∞.

When s→0, Equation (A4) becomes:

(A5) $$\frac{X_{2}}{X_{1}}=\frac{A_{1}}{A_{2}}\frac{k}{\left[k+{\left(\frac{A_{c}}{A_{2}}\right)}^{2}k_{2}\right]}$$

If we assume the outlet of the channel is opened to the atmosphere, which gives $k_{2}=0$, the transfer function becomes $X_{2}/X_{1}=A_{1}/A_{2}$. It means the gain of the function only depends on the spatial ratio between the cross-sectional areas. It is physically reasonable, because the gain at the frequency approaching to zero has nothing to do with temporal elements, such as the dampers in Figure 2, and the pushed in fluid would fully transform to the movement of the object. On the other hand, if we assume the spring on the outlet $k_{2}\to \infty$, it means that the outlet is rigid and no fluid can be pushed into the channel. As a result, the gain of the manipulation becomes $X_{2}/X_{1}=0$. That means, all the movement of the macro actuator would only result in the motion of PDMS chip deformation, $x_{c}$.

When the input frequency is infinity s→∞, the response becomes:

(A6) $$\frac{X_{2}}{X_{1}}=\frac{A_{1}}{A_{2}}\frac{M}{\left[M+{\left(\frac{A_{c}}{A_{2}}\right)}^{2}m\right]}$$

In typical microfluidic modeling, the inertia of the fluid in the microchannel is neglected as m=0 and, thus, the response also only depends on the ratio between the cross-sectional areas as $x_{2}/x_{1}=A_{1}/A_{2}$. This result can be understood as that the PDMS walls as low-pass filters, and behave like rigid walls when the frequency is very high. For a more general solution without assuming m=0, a greater inertia m of the fluid in the channel would lead to a smaller gain.

Based on Equation (4), the zeros and poles of the transfer function are defined as:

(A7) $$\text{Zeros: }\omega_{1}\left(-\zeta_{1}\pm \sqrt{\zeta_{1}^{2}-1}\right)$$

(A8) $$\text{Poles: }\omega_{2}\left(-\zeta_{2}\pm \sqrt{\zeta_{2}^{2}-1}\right)$$

When the manipulation frequency hits the zeros, no motion of the micro object will be detected since the gain is zero as the numerator in Equation (4) equals zero. On the other hand, the gain will be out of control if the manipulation frequency hits the poles, which makes the denominator of Equation (4) zero. Equation (4) will later be used to perform the numerical fitting for determining the parameters from experimental results.

The response of a sinusoidal input can be calculated by letting s=jω, which gives:

(A9) $$G\left(j\omega \right)=\alpha \frac{\left(\omega_{1}^{2}-\omega^{2}\right)+j\left(2\zeta_{1}\omega_{1}\omega \right)}{\left(\omega_{2}^{2}-\omega^{2}\right)+j\left(2\zeta_{2}\omega_{2}\omega \right)}$$

The gain can then be derived as:

(A10) $$\left|G\left(jw\right)\right|=\left|\alpha \right|\frac{\left|\left(\omega_{1}^{2}-\omega^{2}\right)+j\left(2\zeta_{1}\omega_{1}\omega \right)\right|}{\left|\left(\omega_{2}^{2}-\omega^{2}\right)+j\left(2\zeta_{2}\omega_{2}\omega \right)\right|}=\alpha \frac{\sqrt{\omega^{4}+\left({\left(2\zeta_{1}^{2}\omega_{1}\right)}^{2}-2\omega_{1}^{2}\right)\omega^{2}+\omega_{1}^{4}}}{\sqrt{\omega^{4}+\left({\left(2\zeta_{2}^{2}\omega_{2}\right)}^{2}-2\omega_{2}^{2}\right)\omega^{2}+\omega_{2}^{4}}}$$

## Appendix B

In order to obtain the frequency responses of the system, such as phase and gain, the experimental results, as the input and output signals are plugged into FFT for performing spectrum analysis. A series of complex values with respect to frequencies can be obtained after FFT. The gain and phase of them can be calculated as their distance and angle from the origin on a complex coordinate. This appendix explains the step-by-step procedure of how the gain and phase is determined using an example of the results at 10 Hz.

Figure A1 shows an example of the FFT results on a complex coordinate while the input frequency is 10 Hz. Each point represents a complex number at a frequency, and the points are connected in the order of frequencies from low to high. The right-half part of Figure A1 shows a zoomed-in view of the dashed box on the left-half graph. It can be seen that the distance, the absolute value of complex values, are large at low frequencies and the input frequency 10 Hz. The lower frequency points indicate the component of steady drift of microbeads. It may be due to an unbalanced pressure between the inlet and outlet of the channel, or sometimes it can be caused by secondary flow under high frequencies [24]. On the other hand, the 10 Hz peak indicates the most dominant frequency for the microbeads motion. The gain can be calculated as the amplitudes ratio between the input and output while the phase can be found as the angle between the input and output vectors on the complex coordinate. For example, the phase angle at 10 Hz is indicated as −103.96° in Figure A1. The real phase lag should be compensated with 2 ms system delay, which gives the actual phase lag as −96.76° in this particular case. Through the FFT analysis, we can determine the gain and phase at the frequency we are interested, and filter the other unwanted signals, such as flow drift.
